# Microbial landscape: composition and health associations of environmental microbiome in key functional spaces of premium elderly care facilities

**DOI:** 10.1128/spectrum.01837-25

**Published:** 2026-01-30

**Authors:** Jianlou Yang, Xingsheng Qin, Dianbo Zhang, Chen Dong

**Affiliations:** 1 School of Sport Management, Shandong Sport University 105834, Jinan, Shandong, China; Commonwealth Scientific and Industrial Research Organisation, Brisbane, Australia

**Keywords:** environmental microbiome, elderly care facilities, 16S rRNA sequencing, spatial heterogeneity, antibiotic resistance genes, microbial ecology

## Abstract

The environmental microbiome in elderly care facilities plays a crucial role in the health of aging populations with immunosenescence; however, its composition and health associations remain underexplored. This study characterizes the microbial ecology of premium elderly care facilities, focusing on key functional spaces, environmental drivers, and implications for resident health. We conducted 16S rRNA gene sequencing (V3–V4 regions) on 320 surface and air samples from six functional spaces (dining areas, medical facilities, bedrooms, bathrooms, recreational rooms, and corridors) across four premium elderly care facilities. Environmental parameters (temperature, humidity, CO₂, and occupancy) were measured concurrently. Bioinformatics analysis (QIIME 2, DADA2, and Silva database) and statistical modeling (permutational multivariate analysis of variance, distance-based redundancy analysis, and PICRUSt2) were employed to assess microbial diversity, taxonomic composition, functional potential, and environmental correlations. Using 16S rRNA gene sequencing across four facilities in different geographic regions, we identified significant spatial heterogeneity in microbial diversity and composition, with dining areas and recreational rooms exhibiting higher richness (Shannon index: 6.07 ± 0.37) and human-associated taxa (e.g., Firmicutes), while medical facilities and bathrooms harbored lower diversity but elevated opportunistic pathogens (*Pseudomonas* and *Klebsiella*). Environmental parameters—particularly relative humidity (explaining 13.8% of community variation) and occupancy—strongly influenced the microbial structure. A core microbiome dominated by Proteobacteria, Firmicutes, and Actinobacteria was conserved across facilities, while functional predictions revealed space-specific traits, including predicted enrichment of antibiotic resistance genes in medical facilities (ARG Shannon diversity: 4.87 ± 0.42) and carbohydrate metabolism pathways in dining areas. Negative correlations between beneficial (*Lactobacillus*) and pathogenic taxa (*Staphylococcus aureus*) were consistent with potential ecological strategies for microbial balance, although validation with absolute quantification is needed. This study highlights the need for space-specific microbial management in elderly care environments, emphasizing humidity control, ventilation, and targeted hygiene to mitigate pathogen risks while preserving beneficial communities. Our findings suggest the potential value of ecologically informed stewardship over pathogen-centric approaches. Future research should integrate multi-omics and longitudinal health data to optimize microbiome-resident health interactions.

**IMPORTANCE:**

As people age, their immune systems weaken, making the elderly especially vulnerable to germs in their surroundings. This study reveals that the types and amounts of bacteria living on surfaces and in the air within premium elderly care facilities differ significantly depending on the room's purpose—such as dining areas, medical rooms, or bathrooms. We found that humidity and how many people use a space strongly influence these bacterial communities. Crucially, areas like medical rooms had more bacteria linked to infections and antibiotic resistance, while social spaces hosted more diverse and potentially beneficial bacteria. This shows that a “one-size-fits-all” cleaning approach is not ideal. Instead, tailoring hygiene practices and environmental controls (like managing humidity) to specific spaces could better protect residents' health by reducing harmful germs while supporting helpful ones, offering a smarter way to manage these critical living environments for our aging population.

## INTRODUCTION

The global demographic shift toward aging populations has led to a substantial increase in elderly care facilities worldwide (1, 2). In China, the proportion of citizens aged 65 years and older has reached 14.9% in 2023, with projections indicating that this figure will exceed 30% by 2050 (3). Consequently, premium elderly care facilities have emerged as specialized living environments designed to optimize the quality of life for aging populations. These environments represent unique ecological niches where elderly residents, who often have compromised immune systems and specific health vulnerabilities, interact with environmental microbiomes for extended periods (4, 5).

Environmental microbiomes in built environments significantly impact human health, particularly for vulnerable populations such as the elderly (6, 7). Recent advances in next-generation sequencing technologies have enabled a comprehensive characterization of microbial communities in various built environments, including hospitals (8), schools (9), and residential buildings (10). However, there remains a notable knowledge gap regarding the microbial ecology of elderly care facilities, especially premium facilities with specialized functional spaces (11, 12). This gap is particularly significant considering that elderly individuals typically spend 80%–90% of their time indoors and may have altered microbiome interactions due to age-related immunosenescence (13, 14).

The functional spaces within elderly care facilities—such as dining areas, recreational rooms, medical facilities, and bedrooms—serve distinct purposes and potentially harbor unique microbial communities shaped by specific uses, occupancy patterns, and cleaning protocols (15). These spaces may act as reservoirs for various microorganisms, including beneficial commensals, opportunistic pathogens, and microbes with potential health-modulating effects (16, 17). Understanding the composition and dynamics of these microbial communities is crucial for developing evidence-based strategies to create healthier living environments for elderly populations (18).

Previous studies have primarily focused on healthcare-associated infections in nursing homes (19, 20) or specific pathogenic microorganisms in elderly care facilities (21). However, comprehensive characterization of the entire microbial community across different functional spaces in premium elderly care facilities remains unexplored. The transition from pathogen-centric approaches to ecosystem-based perspectives represents a paradigm shift in understanding environmental health in elderly care settings (22, 23).

In this study, we employed 16S rRNA gene sequencing to characterize the environmental microbiome across key functional spaces in four premium elderly care facilities in Jinan, Qingdao, Fuzhou, and Shanghai, China. Our research addresses the following three main questions. (i) What are the compositional characteristics and diversity patterns of microbial communities in different functional spaces of premium elderly care facilities? (ii) How do environmental factors influence the structure and diversity of these microbial communities? (iii) What are the potential implications of these microbial profiles for elderly health and facility management?

By addressing these questions, our study provides novel insights into the microbial ecology of premium elderly care environments, contributing to the development of microbially informed design and management practices that can potentially enhance the health and well-being of elderly residents.

## MATERIALS AND METHODS

### Study design and sampling sites

Four premium elderly care facilities (designated as ECF-A, ECF-B, ECF-C, and ECF-D) in Jinan, Qingdao, Fuzhou, and Shanghai, China, were selected for this study based on their service quality ratings, occupancy rates (>85%), and operational history (>3 years). For the purposes of this study, “premium” facilities were operationally defined by the following criteria: (i) monthly costs exceeding 5,000 renminbi per resident (approximately 2–3 times the regional average for standard nursing homes); (ii) infrastructure meeting or exceeding national Class A standards for elderly care facilities (24), including individual heating, ventilation, and air conditioning (HVAC) systems, mechanical ventilation with fresh air exchange rates ≥30 m³/h per person, and medical-grade air filtration systems; (iii) staffing ratios of at least 1:3 (staff to residents), compared to typical ratios of 1:6–1:10 in standard facilities; (iv) standardized cleaning protocols with daily disinfection of high-touch surfaces using hospital-grade disinfectants and weekly deep cleaning; and (v) on-site medical facilities staffed by licensed healthcare professionals. These characteristics distinguish premium facilities from standard nursing homes in terms of environmental control, hygiene management, and resource allocation, which may influence microbial community composition. Each facility accommodated between 120 and 180 residents, with an average age of 78.5 ± 6.2 years. The facilities provided comprehensive services including healthcare, rehabilitation, dietary management, and recreational activities. All facilities maintained similar environmental conditions (temperature: 22°C–26°C and relative humidity: 40%–60%) and implemented standardized cleaning protocols according to national elderly care standards (24).

### Environmental sampling

Surface samples were collected using sterile nylon flocked swabs (Copan Diagnostics, Italy) pre-moistened with sterile 0.15 M NaCl solution with 0.1% Tween 20 (25). For each functional space, 10 sampling points were selected based on high-touch surfaces and areas with frequent human contact (26). Each sampling point covered approximately 100 cm² using the swabbing protocol described by Adams et al. (27). Swabs were immediately placed in sterile tubes containing 2 mL of RNAlater solution (Thermo Fisher Scientific, USA) and transported to the laboratory on ice within 4 h of collection.

Indoor air samples were collected using a BioSampler SKC (SKC Inc., USA) at a flow rate of 12.5 L/min for 30 min in each functional space (28). The sampler was placed at a height of 1.5 m to represent the breathing zone. Air samples were collected into 20 mL of phosphate-buffered saline (PBS) with 0.005% Tween 80. Upon completion of sampling, the liquid was transferred to sterile 50 mL centrifuge tubes and transported to the laboratory on ice.

Environmental parameters, including temperature, relative humidity, carbon dioxide (CO_2_) concentration, particulate matter (PM_2.5_ and PM_10_) levels, and occupancy counts, were measured simultaneously with microbial sampling using calibrated portable devices (TSI Q-Trak 7575, USA; Dusttrak DRX 8534, USA). These parameters were recorded at 5-min intervals throughout the 30-min sampling period (29).

### DNA extraction and 16S rRNA gene sequencing

#### Sample processing prior to DNA extraction

For surface swab samples, the swabs stored in 2 mL RNAlater solution were first vortexed vigorously for 60 s to release microbial cells from the swab fibers into the liquid. The swab heads were then pressed against the inner wall of the tube while rotating to maximize cell recovery. The swabs were subsequently removed, and the liquid was transferred to sterile 2 mL microcentrifuge tubes. Samples were centrifuged at 10,000 × *g* for 10 min at 4°C to pellet microbial cells. The supernatant was carefully removed, leaving approximately 100 μL of liquid with the cell pellet to prevent sample loss. For air samples collected in PBS, the 20 mL liquid samples were first concentrated by centrifugation at 5,000 × *g* for 15 min at 4°C. The supernatant was discarded, retaining only the pellet and approximately 200 μL of residual liquid. If the pellet was not visible, the sample was subjected to a second centrifugation step at 10,000 × *g* for 10 min. The concentrated pellets from both surface and air samples were then resuspended in 200 μL of PBS by gentle pipetting before proceeding to DNA extraction.

#### DNA extraction protocol

Total genomic DNA was extracted from the concentrated swab and air samples using the DNeasy PowerSoil Pro Kit (Qiagen, Germany) following the manufacturer’s instructions with minor modifications as described below. Briefly, the 200 μL resuspended cell pellet was added to the PowerBead Pro Tube containing ceramic beads provided in the kit. An additional 550 μL of CD1 solution (lysis buffer) was added to each tube. Samples were subjected to bead-beating using a FastPrep-24 5G instrument (MP Biomedicals, USA) at 6.0 m/s for 45 s, followed by a 5-min incubation at room temperature. This bead-beating step was repeated once to ensure thorough cell lysis, particularly for gram-positive bacteria with robust cell walls.

Following bead-beating, the samples were briefly centrifuged at 15,000 × *g* for 1 min to pellet beads and debris. Up to 600 μL of the supernatant was transferred to a clean 2 mL microcentrifuge tube. Then, 200 μL of CD2 solution (inhibitor removal solution) was added, and the samples were vortexed for 5 s before incubation at 4°C for 10 min to precipitate inhibitors. Samples were centrifuged at 15,000 × *g* for 2 min, and up to 750 μL of supernatant was transferred to a new 2 mL microcentrifuge tube, avoiding the pellet.

Subsequently, 650 μL of CD3 solution (binding buffer) was added to the supernatant and mixed by vortexing for 5 s. The entire sample (approximately 1,400 μL) was loaded onto a MB Spin Column in two sequential applications, with centrifugation at 15,000 × *g* for 1 min between loadings. The flow-through was discarded after each centrifugation.

The MB Spin Column was washed twice: first with 500 μL of EA1 solution (wash buffer 1) and then with 500 μL of EA2 solution (wash buffer 2), with centrifugation at 15,000 × *g* for 1 min after each wash. After the final wash, the column was centrifuged for an additional 2 min at 15,000 × *g* to remove residual ethanol from the wash buffers.

Finally, the MB Spin Column was placed in a clean 2 mL microcentrifuge tube, and 50 μL of EB solution (elution buffer, prewarmed to 56°C) was applied directly to the center of the membrane. The column was incubated at room temperature for 5 min to maximize DNA yield and then centrifuged at 15,000 × *g* for 1 min to elute the DNA. This elution step was repeated once with an additional 50 μL of EB solution to maximize DNA recovery, resulting in a final elution volume of approximately 100 μL.

DNA concentration and purity were assessed using a NanoDrop 2000 spectrophotometer (Thermo Fisher Scientific, USA) and Qubit 4.0 Fluorometer with the dsDNA HS Assay Kit (Invitrogen, USA). Only samples with DNA concentrations >5 ng/µL and A260/A280 ratios between 1.8 and 2.0 were used for subsequent analysis. Extracted DNA was stored at −80°C until PCR amplification. To minimize freeze-thaw cycles, DNA samples were aliquoted into multiple tubes (10 μL per tube) for PCR setup.

The V3–V4 hypervariable regions of the bacterial 16S rRNA gene were amplified using the universal primers 341F (5′-CCTACGGGNGGCWGCAG-3′) and 805R (5′-GACTACHVGGGTATCTAATCC-3′) (30). It should be noted that while the 341F/805R primer pair provides broad coverage of bacterial diversity, certain taxa within Proteobacteria (e.g., some SAR11 clade members) and Firmicutes (e.g., certain Clostridiales) may be underrepresented due to mismatches in primer binding sites (30). This primer bias, common to all 16S rRNA-based approaches, may influence the completeness of taxonomic profiles and, consequently, the accuracy of downstream functional predictions based on these profiles. PCR amplification was performed in triplicate for each sample in a 25 μL reaction mixture containing 12.5 μL of 2× Phusion High-Fidelity PCR Master Mix (New England Biolabs, USA), 1 μL of each primer (10 μM), 2 μL of template DNA, and 8.5 μL of nuclease-free water. The thermal cycling conditions were as follows: initial denaturation at 98°C for 30 s; 30 cycles of denaturation at 98°C for 10 s, annealing at 55°C for 30 s, and extension at 72°C for 30 s; and a final extension at 72°C for 5 min (31).

The triplicate PCR products were pooled, purified using the GeneJET Gel Extraction Kit (Thermo Fisher Scientific, USA), and quantified using the Qubit 4.0 Fluorometer. Equimolar amounts of purified amplicons were pooled and paired-end sequenced (2 × 300 bp) on an Illumina MiSeq platform (Illumina Inc., USA) according to standard protocols (32).

### Bioinformatic analysis

Raw sequencing data were processed using the QIIME 2 pipeline (version 2023.5) (33). After demultiplexing, the sequences were quality-filtered, denoised, merged, and chimera-filtered using the DADA2 plugin (34) with the following parameters: truncation length of 270 bp and 210 bp for forward and reverse reads, respectively; maximum expected error of 2.0; and truncation quality score threshold of 25. The resulting amplicon sequence variants (ASVs) were taxonomically classified using the Silva database (release 138.1) with a confidence threshold of 0.8 (35).

Alpha diversity metrics, including observed ASVs, Shannon diversity index, Faith’s phylogenetic diversity, and Pielou’s evenness, were calculated using the q2-diversity plugin in QIIME 2. Beta diversity was assessed using weighted and unweighted UniFrac distances (36) and visualized using principal coordinates analysis (PCoA). To identify differentially abundant taxa between functional spaces, analysis of composition of microbiomes (ANCOM) was implemented in QIIME 2 with a W-statistic cutoff of 0.7 (37).

Functional profiles of the microbial communities were predicted using PICRUSt2 (38) with default parameters. The predicted gene families were categorized into KEGG Orthology (KO) groups and pathways. Additionally, potential pathogenic bacteria were identified through taxonomic assignment and comparison with the Virulence Factor Database (VFDB) (39).

### Statistical analysis

All statistical analyses were performed in R (version 4.2.1) (40) using the phyloseq (41), vegan (42), and DESeq2 (43) packages. Differences in alpha diversity metrics among facility types and functional spaces were evaluated using one-way ANOVA, followed by Tukey’s honestly significant difference (HSD) post-hoc test for normally distributed data or Kruskal-Wallis followed by Dunn’s test for non-normally distributed data. Normality was assessed using the Shapiro-Wilk test.

Permutational multivariate analysis of variance (PERMANOVA) with 999 permutations was used to test the significance of differences in microbial community composition between facility types and functional spaces (44). Distance-based redundancy analysis (db-RDA) was applied to examine the relationships between environmental parameters and microbial community structure (45). Spearman’s rank correlation analysis was used to assess associations between specific taxa and environmental parameters. *P*-values were adjusted for multiple comparisons using the Benjamini-Hochberg procedure, with adjusted *P* < 0.05 considered statistically significant.

Network analysis was performed using the SPIEC-EASI (SParse InversE Covariance Estimation for Ecological Association Inference) method (46) to identify significant co-occurrence relationships between microbial taxa. Network properties, including modularity, connectivity, and centrality measures, were calculated using the igraph package in R (47).

## RESULTS

### Overall microbial diversity comparison among four elderly care facilities

**Fig 1:**
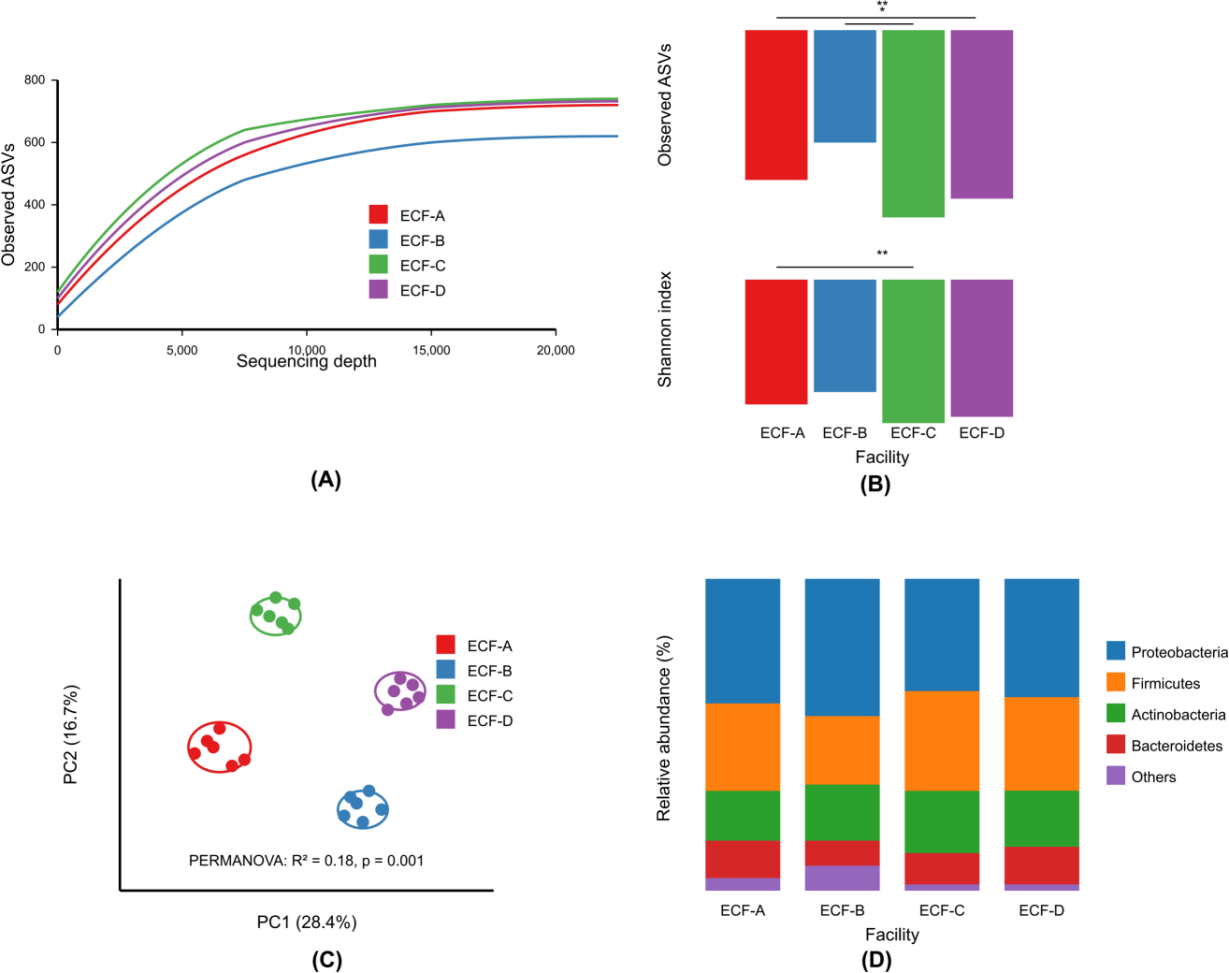
Overall microbial diversity comparison among four elderly care facilities. (**A**) Rarefaction curves. (**B**) Alpha diversity metrics. (**C**) PCoA of weighted UniFrac distances. (**D**) Phylum-level composition.

The environmental microbiome sequencing analysis yielded a total of 3,842,156 high-quality sequences across all samples, resulting in 9,873 ASVs after quality filtering and chimera removal. Rarefaction curves reached saturation at approximately 15,000 sequences per sample, indicating sufficient sequencing depth to capture the microbial diversity (Fig. 1A).

Alpha diversity metrics revealed significant differences in microbial community richness and diversity among the four elderly care facilities (ECF-A, ECF-B, ECF-C, and ECF-D) (Fig. 1B). ECF-C exhibited the highest microbial richness (observed ASVs: 482.3 ± 56.7) and Shannon diversity index (5.84 ± 0.41), followed by ECF-D (observed ASVs: 437.8 ± 62.3; Shannon index: 5.62 ± 0.38), ECF-A (observed ASVs: 398.5 ± 48.2; Shannon index: 5.43 ± 0.45), and ECF-B (observed ASVs: 356.9 ± 53.8; Shannon index: 5.21 ± 0.53) (ANOVA, *P* < 0.01 for both metrics). Faith’s phylogenetic diversity showed a similar pattern, with ECF-C displaying the highest values (42.7 ± 4.1), significantly different from ECF-B (35.8 ± 3.9) (Tukey’s HSD, adjusted *P* < 0.05).

Beta diversity analysis using weighted UniFrac distances revealed distinct clustering patterns among the four facilities (Fig. 1C). PERMANOVA analysis confirmed significant differences in microbial community composition between facilities (R^2^ = 0.18, *P* = 0.001). Pairwise comparisons revealed that all facilities differed significantly from each other (adjusted *P* < 0.05), with the greatest dissimilarity observed between ECF-B and ECF-C (R^2^ = 0.24, *P* = 0.001).

Despite these differences, all facilities shared a common core microbiome at the phylum level, dominated by Proteobacteria (38.4%–45.7%), Firmicutes (21.3%–28.5%), Actinobacteria (12.6%–18.1%), and Bacteroidetes (8.2%–12.3%), which collectively accounted for approximately 85% of the total microbial community (Fig. 1D).

Given that the four facilities were located in cities spanning different geographic and climatic zones (Jinan: temperate continental climate; Qingdao: temperate monsoon climate with maritime influence; Fuzhou: subtropical monsoon climate; and Shanghai: subtropical humid climate), we performed additional analyses to assess potential regional effects on microbial community composition. PERMANOVA analysis revealed significant but modest differences in microbial communities among cities (R² = 0.12, *P* = 0.002), which was considerably lower than the variation explained by functional space types (R² = 0.26, *P* = 0.001) described in subsequent sections. Pairwise comparisons showed that facilities in Fuzhou (subtropical) exhibited slightly higher relative abundance of thermophilic taxa compared to facilities in Qingdao and Jinan (temperate zones) (adjusted *P* < 0.05), likely reflecting regional temperature differences. However, the core microbiome composition at the phylum level remained consistent across all four cities, with no city-specific phyla detected. These findings suggest that while geographic location and outdoor climate exert some influence on indoor microbial communities, the functional characteristics of indoor spaces represent the dominant driver of microbial community structure in elderly care facilities, consistent with previous studies showing that building function and occupancy patterns override regional biogeography in shaping indoor microbiomes (15, 48, 49).

### Microbial compositional characteristics and differences across functional spaces

**Fig 2:**
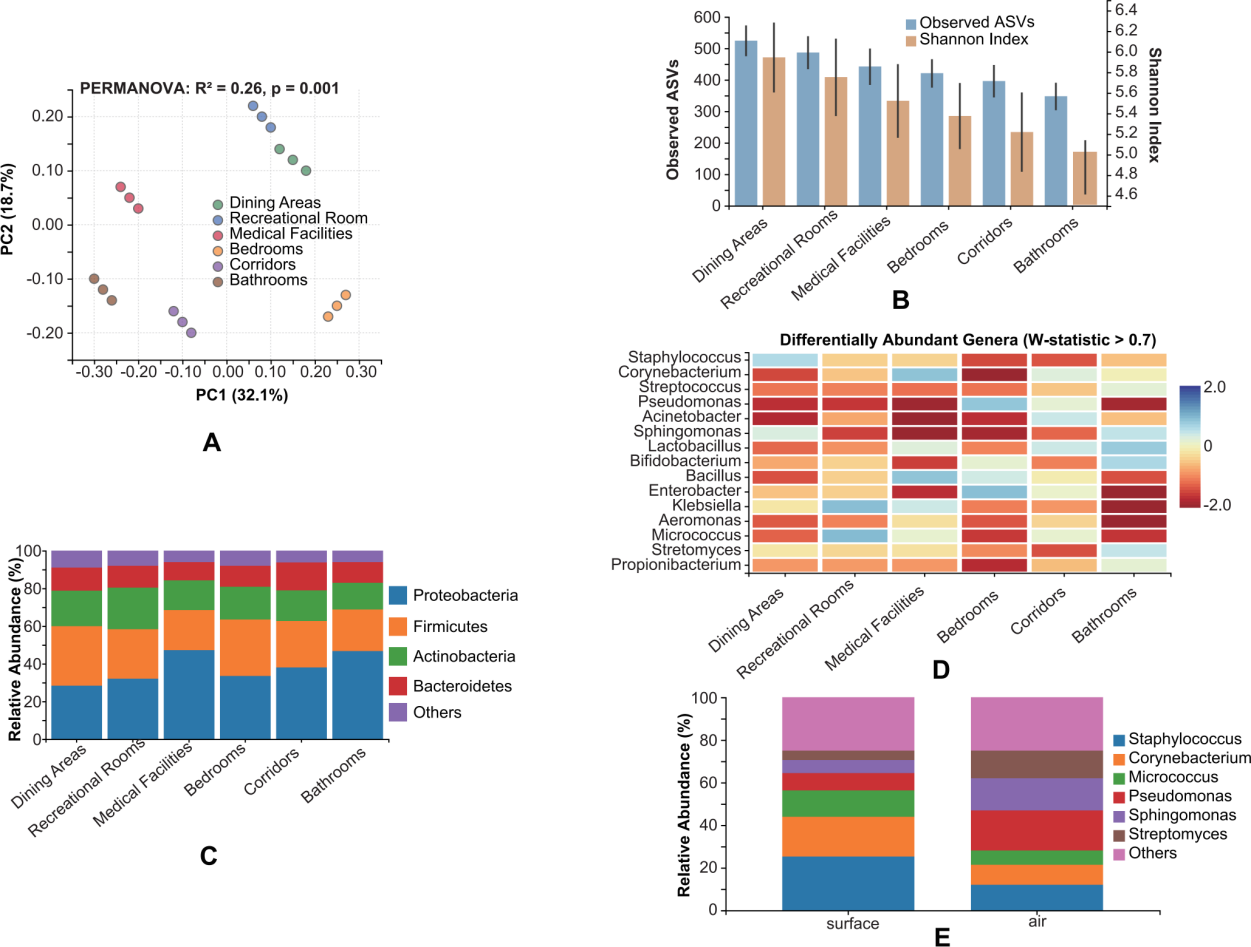
Microbial compositional characteristics and differences across functional spaces. (**A**) Principal coordinate analysis (PCoA) of microbial communities. (**B**) Alpha diversity metrics across functional spaces. (**C**) Phylum-level taxonomic composition. (**D**) Differentially abundant genera (ANCOM analysis). (**E**) Surface vs air sample comparison.

Microbial community structure exhibited significant variations across the six functional spaces (dining areas, recreational rooms, medical facilities, bedrooms, corridors, and bathrooms) within the elderly care facilities (PERMANOVA, R² = 0.26, *P* = 0.001) (Fig. 2A). This spatial heterogeneity was more pronounced than the differences observed between facilities or geographic locations (city effect: R² = 0.12, *P* = 0.002), indicating that space functionality had a stronger influence on microbial composition than either facility-specific factors or regional biogeography.

The dining areas displayed the highest alpha diversity metrics (observed ASVs: 524.1 ± 48.9; Shannon index: 6.07 ± 0.37), followed by recreational rooms (observed ASVs: 486.3 ± 52.4; Shannon index: 5.86 ± 0.41) and medical facilities (observed ASVs: 441.7 ± 57.8; Shannon index: 5.61 ± 0.39). Bathrooms exhibited the lowest diversity (observed ASVs: 347.2 ± 43.6; Shannon index: 5.03 ± 0.47) (Fig. 2B). Significant differences were observed between dining areas and bathrooms for all alpha diversity metrics (Tukey’s HSD, adjusted *P* < 0.01).

Taxonomic analysis at the phylum level revealed distinct compositional patterns across functional spaces (Fig. 2C). Proteobacteria dominated in medical facilities (47.3% ± 5.1%) and bathrooms (46.8% ± 4.8%), while Firmicutes were more abundant in dining areas (31.5% ± 3.7%) and bedrooms (29.8% ± 4.2%). Actinobacteria showed the highest relative abundance in recreational rooms (22.1% ± 2.9%), and Bacteroidetes were most prevalent in corridors (14.8% ± 2.3%).

At the genus level, ANCOM analysis identified 28 differentially abundant taxa across functional spaces (W-statistic > 0.7, Fig. 2D). Notably, *Staphylococcus*, *Corynebacterium*, and *Streptococcus* were significantly enriched in bedrooms (adjusted *P* < 0.01), while *Pseudomonas*, *Acinetobacter*, and *Sphingomonas* dominated in medical facilities (adjusted *P* < 0.01). Dining areas showed higher abundance of *Lactobacillus*, *Bifidobacterium*, and *Bacillus* (adjusted *P* < 0.05), whereas bathrooms were characterized by elevated levels of *Enterobacter*, *Klebsiella*, and *Aeromonas* (adjusted *P* < 0.01).

Surface and air samples exhibited distinct microbial profiles within each functional space (Fig. 2E). Surface samples contained higher proportions of skin-associated genera such as *Staphylococcus*, *Corynebacterium*, and *Micrococcus*, while air samples were enriched with environmental genera including *Pseudomonas*, *Sphingomonas*, and *Streptomyces*. This pattern was consistent across all functional spaces, suggesting different sources and dispersal mechanisms for surface and airborne microbiota.

### Core microbiome identification and functional prediction

**Fig 3:**
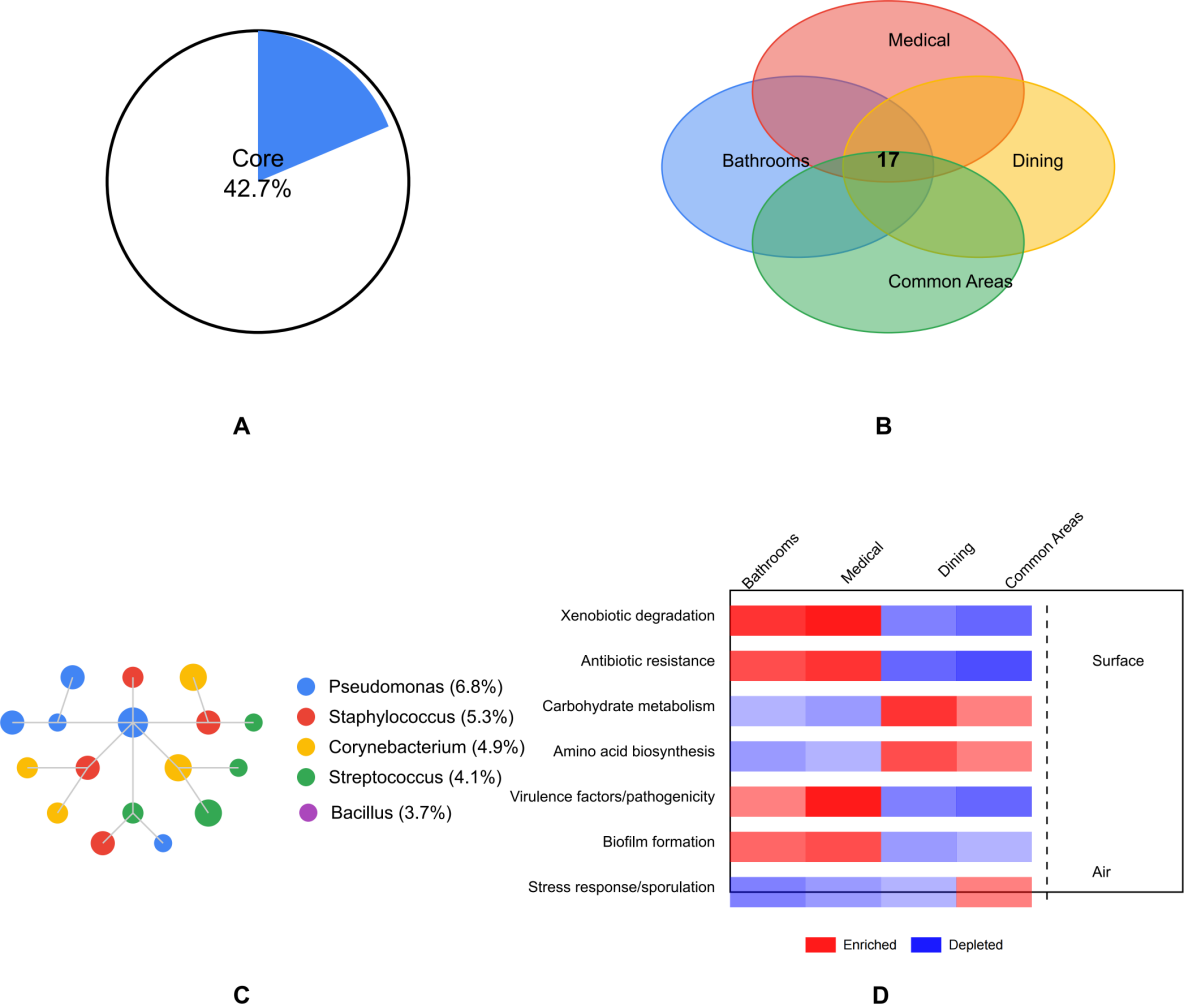
Core microbiome identification and functional prediction. (**A**) Core microbiome (83 ASVs). (**B**) Network of core taxa (376 connections). (**C**) Shared core taxa across functional spaces. (**D**) Predicted functional pathways.

Network analysis identified 83 ASVs as the core microbiome (present in >75% of all samples with relative abundance >0.1%), representing approximately 42.7% of the total microbial community (Fig. 3A). These core taxa formed a highly connected network with 376 significant co-occurrence relationships (Fig. 3B). The network exhibited a modular structure (modularity = 0.42) with five distinct clusters, suggesting potential ecological interactions and niche partitioning within the microbial community.

The core microbiome was dominated by genera known to be widespread in built environments, including *Pseudomonas* (6.8%), *Staphylococcus* (5.3%), *Corynebacterium* (4.9%), *Streptococcus* (4.1%), and *Bacillus* (3.7%). Each functional space harbored a distinct subset of core taxa, with 17 ASVs shared across all spaces, representing a facility-wide core microbiome (Fig. 3C).

Functional prediction using PICRUSt2 revealed significant differences in the predicted metabolic potential across functional spaces (Fig. 3D). Based on taxonomic composition, the microbial communities in bathrooms and medical facilities were predicted to have higher relative abundance of genes related to xenobiotic degradation (KEGG pathway ko01220) and antibiotic resistance (KEGG pathway ko01501) (adjusted *P* < 0.01). Dining areas displayed higher abundance of genes involved in carbohydrate metabolism (KEGG pathway ko01200) and amino acid biosynthesis (KEGG pathway ko01230) (adjusted *P* < 0.05). Notably, medical facilities were predicted to harbor microbial communities with the highest proportion of genes associated with virulence factors and pathogenicity (KEGG pathway ko05111) (adjusted *P* < 0.01). It is important to note that these predictions represent the functional potential of the microbial communities based on reference genomes and do not confirm the actual expression or functional activity of these resistance genes.

Significant differences were also observed in the predicted functional capacity between surface and air microbiomes. Surface microbiomes were predicted to show enrichment in genes related to biofilm formation (KEGG pathway ko05111) and antimicrobial resistance (KEGG pathway ko01501), while air microbiomes were predicted to be characterized by higher abundance of genes involved in stress response (KEGG pathway ko04010) and sporulation (KEGG pathway ko02020) (adjusted *P* < 0.01).

### Correlation between environmental parameters and microbial composition

**Fig 4:**
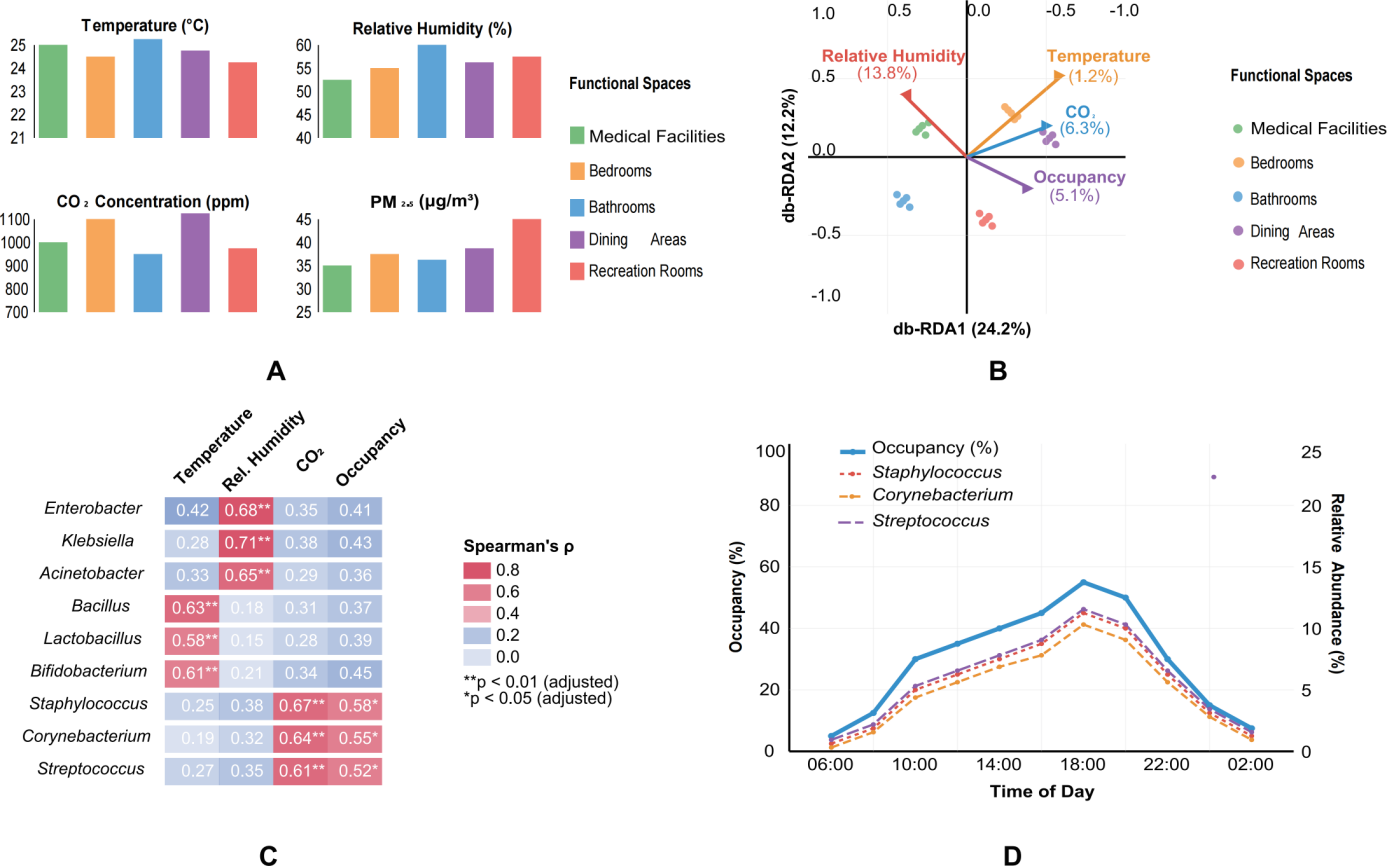
Correlation between environmental parameters and microbial composition across functional spaces. (**A**) Environmental parameters by functional space. (**B**) Distance-based redundancy analysis (db-RDA). (**C**) Spearman’s correlation between environmental parameters and microbial taxa. (**D**) Temporal variations in occupancy and human-associated taxa.

Environmental parameters varied significantly across functional spaces (Fig. 4A). Medical facilities maintained the lowest temperature (22.4°C ± 0.8°C) and relative humidity (42.3% ± 3.5%), while bathrooms exhibited the highest humidity levels (58.7% ± 4.2%). CO₂ concentrations were highest in bedrooms (972.6 ± 86.3 ppm) and dining areas during mealtimes (1,023.5 ± 112.8 ppm). PM₂.₅ levels were elevated in recreational rooms (38.6 ± 5.7 μg/m³) compared to other spaces (Kruskal-Wallis, *P* < 0.01).

db-RDA revealed that environmental parameters explained approximately 36.4% of the total variation in microbial community composition (Fig. 4B). Relative humidity and temperature emerged as the strongest predictors (13.8% and 11.2% of variation, respectively), followed by CO₂ concentration (6.3%) and occupancy (5.1%) (PERMANOVA, *P* < 0.01 for all parameters).

Spearman’s correlation analysis identified significant associations between specific environmental parameters and microbial taxa (Fig. 4C). Relative humidity showed significant positive correlations with the relative abundance of *Enterobacter*, *Klebsiella*, and *Acinetobacter* (Spearman’s ρ > 0.65, adjusted *P* < 0.01), while temperature was positively correlated with *Bacillus*, *Lactobacillus*, and *Bifidobacterium* (Spearman’s ρ > 0.58, adjusted *P* < 0.01). These associations suggest, but do not prove, that these environmental parameters influence the abundance of specific taxa. CO₂ concentration showed strong positive correlations with human-associated genera such as *Staphylococcus*, *Corynebacterium*, and *Streptococcus* (Spearman’s ρ > 0.61, adjusted *P* < 0.01).

Occupancy patterns also significantly influenced microbial community structure. Spaces with higher occupancy rates showed increased alpha diversity (Shannon index; Spearman’s ρ = 0.72, adjusted *P* < 0.001) and elevated relative abundance of human-associated taxa. Temporal variations in occupancy throughout the day correlated with fluctuations in the relative abundance of several human-associated genera, including *Staphylococcus*, *Streptococcus*, and *Corynebacterium* (Fig. 4D).

### Distribution patterns of potentially beneficial bacteria and opportunistic pathogens

**Fig 5:**
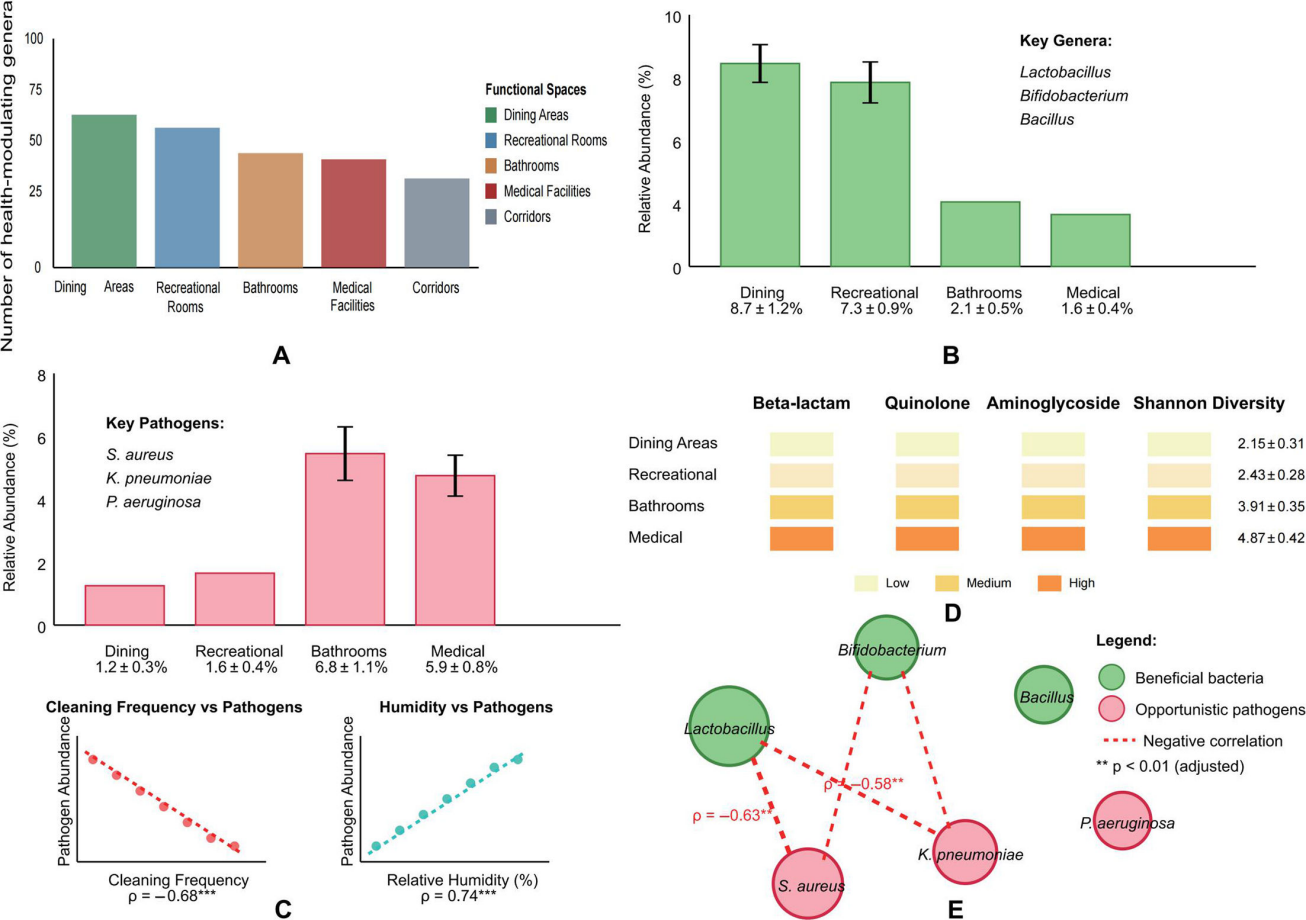
Distribution patterns of potentially beneficial bacteria and opportunistic pathogens. (**A**) Health-modulating genera distribution. (**B**) Potentially beneficial bacteria. (**C**) Opportunistic pathogens. (**D**) Antibiotic resistance gene (ARG) distribution. (**E**) Co-occurrence patterns between beneficial bacteria and pathogens.

Analysis of the taxonomic profiles identified 37 genera with known health-modulating properties, including potentially beneficial bacteria and opportunistic pathogens (Fig. 5A). The relative abundance and distribution of these taxa varied significantly across functional spaces (PERMANOVA, R^2^ = 0.31, *P* = 0.001).

Potentially beneficial bacteria, including *Lactobacillus*, *Bifidobacterium*, and *Bacillus*, were predominantly found in dining areas (collective relative abundance: 8.7% ± 1.2%) and recreational rooms (7.3% ± 0.9%) (Fig. 5B). These genera are known for their probiotic properties and potential health benefits for elderly populations. *Lactobacillus* species were particularly abundant on dining tables and food preparation surfaces (relative abundance: 4.2% ± 0.7%).

In contrast, opportunistic pathogens such as *Staphylococcus aureus*, *Klebsiella pneumoniae*, and *Pseudomonas aeruginosa* (identified through species-level classification and comparison with the VFDB) were more prevalent in bathrooms (collective relative abundance: 6.8% ± 1.1%) and medical facilities (5.9% ± 0.8%) (Fig. 5C). Notably, the relative abundance of these opportunistic pathogens showed significant negative correlation with cleaning frequency (Spearman’s ρ = −0.68, adjusted *P* < 0.001) and positively correlated with relative humidity (Spearman’s ρ = 0.74, adjusted *P* < 0.001). While these correlations are strong, the observational nature of our study precludes causal inference.

The predicted presence and diversity of antibiotic resistance genes (ARGs) inferred from PICRUSt2 analysis showed varying distribution patterns across functional spaces (Fig. 5D). Based on the taxonomic composition, medical facilities were predicted to harbor the highest diversity and abundance of ARGs (Shannon diversity of ARG profiles: 4.87 ± 0.42), particularly genes potentially conferring resistance to beta-lactams, quinolones, and aminoglycosides. It is important to note that these predictions represent the functional potential of the microbial communities based on reference genomes and do not confirm the actual expression or functional activity of these resistance genes. Bathrooms ranked second in ARG abundance, while dining areas exhibited the lowest levels.

The co-occurrence patterns between potentially beneficial bacteria and opportunistic pathogens revealed interesting ecological relationships (Fig. 5E). Significant negative correlations were observed between *Lactobacillus* and *Bifidobacterium* and *S. aureus* (Spearman’s ρ = −0.63, adjusted *P* < 0.01) as well as *K. pneumoniae* (Spearman’s ρ = −0.58, adjusted *P* < 0.01). While these negative correlations are consistent with potential competitive or antagonistic interactions between beneficial and pathogenic bacteria within the built environment of elderly care facilities, it is important to note that such patterns in relative abundance data may also arise from compositional effects rather than genuine biological interactions and would require validation with absolute quantification methods.

## DISCUSSION

This study provides a comprehensive characterization of the environmental microbiome across different functional spaces in premium elderly care facilities, revealing distinct microbial communities shaped by space functionality, environmental parameters, and human occupancy patterns. Our findings contribute to the growing body of knowledge on microbial ecology in built environments and offer insights into potential implications for elderly health and facility management.

### Spatial heterogeneity of microbial communities

The observed spatial heterogeneity in microbial communities across different functional spaces aligns with previous studies on built environments (48, 49). However, our finding that space functionality exerts a stronger influence on microbial composition than facility-specific factors represents a novel contribution to understanding microbial ecology in elderly care settings. This pattern suggests that the activities and purposes associated with each space create unique microbial niches, potentially through differences in human behavior, materials, cleaning protocols, and environmental conditions (50). An important finding of our study is that space functionality exerted a substantially stronger influence on microbial composition (R² = 0.26) than geographic location/city (R² = 0.12), despite the four facilities being located in cities with distinct climatic zones ranging from temperate continental to subtropical climates. This pattern indicates that the microbial ecology of indoor environments in elderly care facilities is primarily shaped by anthropogenic factors—such as space use, occupancy, and management practices—rather than by regional biogeography or outdoor microbial sources (15, 48). The observed consistency in core microbiome composition across geographic regions, combined with pronounced differences across functional spaces within facilities, supports the concept that built environments create unique selective pressures that homogenize microbial communities across geographic scales while differentiating them at functional scales (49, 51). This finding has important implications for facility management, suggesting that standardized, space-specific protocols may be broadly applicable across different geographic regions, although local climate factors (e.g., humidity influenced by regional weather patterns) should still be considered in implementation (52).

The highest microbial diversity observed in dining areas and recreational rooms likely reflects the convergence of diverse microbial sources, including food-associated microorganisms, human-associated taxa from multiple occupants, and environmental species (53). These spaces function as community hubs with high occupancy rates and diverse activities, fostering rich microbial ecosystems. Conversely, the lower diversity in bathrooms and medical facilities may result from more stringent cleaning protocols, antimicrobial surface materials, and environmental conditions that select for specific microbial taxa (54, 55).

The taxonomic composition across functional spaces revealed noteworthy patterns. The predominance of Proteobacteria in medical facilities and bathrooms aligns with previous studies reporting high abundance of this phylum in healthcare environments and moisture-rich settings (56, 57). These environments may select for gram-negative bacteria with higher tolerance to disinfectants and antimicrobial agents commonly used in these spaces (58). The enrichment of *Firmicutes* in dining areas and bedrooms likely reflects human influence, as this phylum includes many human-associated genera such as *Staphylococcus* and *Streptococcus* (59). The higher abundance of Actinobacteria in recreational rooms may be related to the diverse materials present in these spaces, as members of this phylum are known to degrade complex organic compounds (60).

### Environmental drivers of microbial community structure

The significant influence of environmental parameters on microbial community composition underscores the importance of indoor environmental quality in shaping microbial ecosystems. Relative humidity emerged as the strongest predictor of microbial community structure, consistent with previous research demonstrating humidity as a critical factor affecting microbial growth and survival in indoor environments (61, 62). This finding aligns with a continental-scale study of hotel rooms across China, which identified relative humidity and temperature as primary environmental determinants of microbial richness and composition, with humidity showing particularly strong effects on community structure (61). The consistency of humidity effects across different building types (hotels and elderly care facilities) and geographic scales suggests universal principles governing indoor microbial ecology. The positive correlation between relative humidity and opportunistic pathogens such as *Enterobacter*, *Klebsiella*, and *Acinetobacter* is particularly notable, suggesting that moisture control may be an effective strategy for reducing the prevalence of these potentially harmful microorganisms (63). However, it is important to note that our cross-sectional, observational study design does not establish causation, and experimental or intervention studies would be necessary to confirm whether humidity control directly reduces pathogen abundance.

Temperature also significantly influenced microbial community composition, with thermophilic genera like *Bacillus* showing positive associations with higher temperatures. This finding aligns with the known temperature preferences of these microorganisms and suggests the potential for temperature regulation as a microbial management strategy (64), although controlled intervention studies would be needed to establish causal relationships. The correlations between CO₂ concentration and human-associated genera further demonstrate the impact of occupancy on microbial communities, supporting the concept that humans serve as important sources and vectors for microorganisms in built environments (15, 65).

The temporal variations in microbial community structure associated with occupancy patterns reveal the dynamic nature of indoor microbiomes. These findings suggest that microbial communities in elderly care facilities undergo daily fluctuations in response to human activities, a phenomenon previously observed in other built environments (9, 66). This temporal dimension adds complexity to environmental microbiome management and highlights the need for adaptive strategies that account for occupancy patterns.

### Implications of core microbiome and functional potential

The identification of a core microbiome across all functional spaces suggests the existence of a stable microbial community adapted to the general conditions of elderly care facilities. This core community, dominated by widespread environmental and human-associated genera such as *Pseudomonas*, *Staphylococcus*, and *Corynebacterium*, may represent key microbial taxa that persist despite variations in space functionality and environmental conditions (51). The modular structure of the co-occurrence network indicates potential ecological interactions and niche partitioning within this core community, contributing to its stability (67).

The predicted functional profiles revealed significant differences in metabolic potential across functional spaces, providing insights into the ecological roles of these microbial communities. The enrichment of genes related to xenobiotic degradation and antibiotic resistance in bathrooms and medical facilities suggests adaptation to the frequent use of cleaning agents and antimicrobials in these spaces (68). This finding raises concerns about these environments potentially serving as reservoirs for antimicrobial resistance genes, a significant public health issue, particularly in healthcare settings (69).

The higher abundance of genes involved in carbohydrate metabolism and amino acid biosynthesis in dining areas likely reflects adaptation to nutrient-rich conditions from food residues (70). Similarly, the enrichment of biofilm formation genes in surface microbiomes compared to air microbiomes demonstrates adaptation to different lifestyle strategies based on habitat (71). These functional differences highlight the specialized roles that microorganisms play in different microhabitats within elderly care facilities. However, it is crucial to emphasize that these functional predictions are based on taxonomic composition and reference genomes and do not represent direct measurements of gene presence or expression. The predicted presence of antibiotic resistance genes, in particular, does not necessarily indicate functional resistance phenotypes, as gene expression, regulation, and horizontal gene transfer dynamics are not captured by 16S rRNA-based predictions (38, 72). Therefore, while these predictions provide valuable hypotheses about the functional potential of microbial communities, they should be interpreted cautiously and validated through metagenomics, metatranscriptomics, or culture-based approaches in future studies.

### Health implications of environmental microbiome

The distribution patterns of potentially beneficial bacteria and opportunistic pathogens across functional spaces have important implications for elderly health. The higher abundance of potentially beneficial bacteria such as *Lactobacillus* and *Bifidobacterium* in dining areas may contribute to a healthier microbiome environment in spaces where food consumption occurs (73). These bacteria are known for their probiotic properties and may help maintain a balanced microbial ecosystem through competitive exclusion of pathogenic species (74).

Conversely, the higher prevalence of opportunistic pathogens in bathrooms and medical facilities highlights these spaces as potential hotspots for harmful microorganisms (23). The negative correlation between cleaning frequency and the relative abundance of these pathogens is consistent with the hypothesis that regular cleaning may reduce potential health risks. Similarly, the positive correlation with relative humidity suggests that moisture control may complement cleaning protocols to help manage pathogen levels (75). However, these correlational observations do not prove causation, and controlled intervention studies are needed to establish the actual effectiveness of these management strategies in reducing pathogen abundance and associated health risks.

The negative correlations observed between beneficial bacteria and opportunistic pathogens are suggestive of potential ecological interventions to promote healthier indoor microbiomes. For instance, if these negative correlations reflect true competitive or antagonistic interactions, encouraging the growth of beneficial bacteria through environmental management may potentially help suppress pathogen abundance through competitive interactions (76, 77). However, it is critical to acknowledge that our study employed 16S rRNA gene sequencing, which provides relative abundance data rather than absolute cell counts or biomass measurements. Negative correlations in compositional data can arise from mathematical constraints of relative abundances (i.e., the closed sum problem) rather than genuine biological antagonism (67). For example, a dramatic increase in the absolute abundance of one taxon will necessarily decrease the relative abundances of all other taxa, even if their absolute abundances remain unchanged or increase modestly. Therefore, while the observed negative correlations between *Lactobacillus*/*Bifidobacterium* and *S. aureus*/*K. pneumoniae* are consistent with competitive exclusion or antagonistic interactions, they could also represent compositional artifacts. Definitive establishment of antagonistic relationships would require absolute quantification approaches, such as quantitative PCR (qPCR) targeting specific taxa, flow cytometry with microbial cell counts, or culture-based enumeration methods (67). Despite this limitation, the ecological hypothesis that beneficial bacteria may competitively exclude pathogens remains plausible and warrants investigation through experimental validation studies. This approach represents a shift from conventional hygiene practices focused solely on reducing microbial load to strategies that promote balanced microbial communities (78).

The predicted enrichment of antibiotic resistance genes across different functional spaces, with highest inferred abundance in medical facilities, aligns with patterns observed in hospital environments (22). However, it is crucial to emphasize that these functional predictions are based on taxonomic composition and reference genomes and do not represent direct measurements of gene presence or expression. The predicted presence of antibiotic resistance genes, in particular, does not necessarily indicate functional resistance phenotypes, as gene expression, regulation, and horizontal gene transfer dynamics are not captured by 16S rRNA-based predictions (38, 72). Therefore, while these predictions provide valuable hypotheses about the functional potential of microbial communities, they should be interpreted cautiously and validated through metagenomics, metatranscriptomics, or culture-based approaches in future studies. Our findings are further supported by a global urban microbiome study that identified environmental factors (particularly humidity and antimicrobial use) and demographic characteristics as key drivers of ARG distribution in built environments across multiple continents (69). That study’s demonstration of significant spatial heterogeneity in ARG profiles across different urban settings parallels our observation of space-specific ARG enrichment patterns within elderly care facilities. While these predictions suggest potential ARG reservoirs, direct metagenomic sequencing would be necessary to confirm the actual presence and diversity of resistance genes (72). While these predictions suggest potential ARG reservoirs, direct metagenomic sequencing would be necessary to confirm the actual presence and diversity of resistance genes (72). This finding underscores the need for antimicrobial stewardship and targeted cleaning protocols in these areas to minimize the spread of resistance genes. The co-occurrence patterns between antibiotic resistance genes and specific taxa provide insights into potential reservoirs and vectors for these genes within the built environment (79).

### Practical implications for elderly care facility management

Our findings have several practical implications for the design and management of elderly care facilities. The distinct microbial communities associated with different functional spaces suggest that cleaning protocols and environmental management strategies should be space-specific rather than facility-wide (18). For example, based on the strong positive correlations we observed, more stringent humidity control in bathrooms and medical facilities may help reduce the prevalence of moisture-loving opportunistic pathogens, while alternative approaches focusing on microbial balance might be more appropriate for dining areas and recreational rooms. However, intervention studies are needed to confirm the causal effects of these management strategies.

The significant influence of environmental parameters on microbial community structure highlights the importance of indoor environmental quality management. Given the significant correlations between environmental parameters and microbial community structure, maintaining optimal temperature and humidity levels, ensuring adequate ventilation to control CO₂ concentrations, and implementing occupancy-based ventilation strategies may help create healthier microbial environments (52, 80), although these recommendations should be validated through controlled interventions. These approaches align with the concept of "bioinformed design," which incorporates microbial ecology principles into building design and management (81).

The temporal dynamics of microbial communities in response to occupancy patterns suggest that cleaning schedules should be aligned with periods of high human activity (82). Additionally, the use of real-time monitoring of environmental parameters may inform adaptive management strategies that respond to changing conditions throughout the day (83), although the effectiveness of such approaches in modulating microbial communities requires empirical testing. Such approaches would be particularly valuable in spaces with fluctuating occupancy, such as dining areas and recreational rooms.

The potential health implications of environmental microbiomes underscore the need for a balanced approach to hygiene in elderly care facilities. While controlling potentially harmful microorganisms remains important, especially for immunocompromised elderly residents, maintaining diverse and balanced microbial communities may also contribute to health benefits (84, 85). This perspective aligns with recent theoretical advances in indoor microbiome research, which emphasize prevention strategies that promote beneficial microbial communities rather than solely focusing on pathogen elimination (75). Such strategies, including targeted probiotic applications and microbiome-informed ventilation protocols, represent promising approaches for elderly care environments where residents may be particularly susceptible to microbiome-related health effects. This perspective represents a paradigm shift from viewing all microorganisms as threats to recognizing their ecological roles and potential contributions to human health (86).

### Limitations and future directions

Despite the comprehensive nature of this study, several limitations should be acknowledged. First, while 16S rRNA gene sequencing provides valuable information about bacterial community composition, it does not capture other microorganisms such as fungi, viruses, and archaea, which are also important components of indoor microbiomes (87). Future studies should employ metagenomic approaches to characterize the complete microbial community, including non-bacterial members and their functional potential (88).

Furthermore, our reliance on 16S rRNA gene sequencing for microbial community characterization means that all abundance data are compositional (relative abundances) rather than absolute. This creates inherent challenges in interpreting correlations between taxa, particularly negative correlations that we interpreted as potentially indicating antagonistic relationships. The compositional nature of sequencing data imposes a mathematical constraint, whereby all relative abundances must sum to 100%, meaning that changes in one taxon necessarily affect the relative abundances of all others, even in the absence of biological interactions. This “closed sum” problem can generate spurious negative correlations that mimic competitive or antagonistic relationships (67). To definitively establish whether the negative correlations we observed between beneficial bacteria (e.g., *Lactobacillus*) and opportunistic pathogens (e.g., *S. aureus*) represent genuine biological antagonism or compositional artifacts, future studies should incorporate absolute quantification methods such as quantitative PCR, flow cytometry, or spike-in standards during sequencing library preparation. Such approaches would enable differentiation between scenarios where both taxa decline in absolute abundance versus scenarios where one increases while the other remains constant or decreases, thereby clarifying the nature of their ecological relationships (46).

Meanwhile, we acknowledge a methodological limitation regarding the absence of negative controls (e.g., blank swabs, DNA extraction kit blanks, and PCR-negative controls) in our sampling and laboratory procedures. In low-biomass environmental microbiome studies, contamination from reagents, consumables, and laboratory environments represents a significant concern that can introduce exogenous DNA and potentially confound taxonomic assignments (89). Without negative controls, we cannot definitively quantify or exclude potential contamination signals from our data set. This limitation is particularly relevant for low-abundance taxa detected in our samples, as certain genera commonly found in reagent contamination may overlap with genuine environmental microorganisms (15, 27). Future studies should incorporate comprehensive negative controls throughout the sampling, extraction, and sequencing workflow to distinguish genuine environmental signals from potential contamination. Despite this limitation, our focus on high-abundance, ecologically relevant taxa and the observation of consistent patterns across multiple facilities and sample types provide confidence in our major findings regarding spatial heterogeneity and environmental drivers of microbial communities (48, 49).

Second, our study provides a snapshot of microbial communities at specific time points but does not capture long-term temporal dynamics across seasons or years. Longitudinal studies would provide insights into temporal stability and succession patterns of microbial communities in elderly care environments (90). Additionally, incorporating resident health data would enable direct assessment of associations between environmental microbiomes and health outcomes, strengthening the clinical relevance of these findings (91).

Third, while PICRUSt2 provides useful predictions of functional potential, these predictions are based on reference genomes and may not accurately represent the actual functional capabilities of the microbial communities. Several factors further limit the accuracy of functional inference in built environment microbiomes: (i) the underrepresentation of environmental microbial taxa in reference databases, as most reference genomes derive from cultured clinical or soil isolates rather than built environment-adapted strains (72); (ii) the inability of 16S rRNA-based methods to resolve strain-level functional variation, which is particularly relevant for antibiotic resistance genes that often show strain-specific distribution patterns (92); and (iii) primer bias inherent in the 341F/805R primer set, which may underrepresent certain *Proteobacteria* and *Firmicutes* lineages, potentially skewing functional predictions (30). These limitations are especially important when interpreting our ARG predictions, as the actual presence, copy number, and expression of resistance genes cannot be confirmed without direct metagenomic sequencing or culture-based phenotypic testing. Future studies should incorporate metatranscriptomic or metaproteomic approaches to directly measure functional activity rather than inferring it from taxonomic composition (72), alongside metagenomics to validate the presence and diversity of predicted functional genes.

An additional limitation of our study is the exclusive focus on resident-centered functional spaces, without sampling visitor reception areas, staff break rooms, or administrative zones. While our sampling design prioritized spaces where elderly residents spend the majority of their time (80%–90% of daily activities), visitor and staff areas may serve as critical entry points for externally transmitted microbes and could significantly influence the overall microbial ecology of the facilities. High-traffic zones such as main entrances, visitor waiting areas, and staff changing rooms warrant investigation in future studies, as these spaces may harbor distinct microbial communities shaped by outdoor air exchange, transient human traffic, and different cleaning protocols. Understanding the microbial transmission pathways from these external contact points to resident spaces would provide a more comprehensive picture of how environmental microbiomes are established and maintained in elderly care facilities. Future research should incorporate the sampling of these transitional zones, coupled with visitor/staff microbiome profiling and tracking studies, to elucidate the dynamics of microbial introduction and dissemination throughout the facility environment.

Finally, our study was conducted in a specific geographic region and cultural context, which may limit the generalizability of findings to elderly care facilities in other regions with different building designs, management practices, and cultural norms. Comparative studies across different geographic and cultural contexts would enhance our understanding of universal versus context-specific patterns in elderly care facility microbiomes (93).

Future research should explore interventions aimed at promoting healthier indoor microbiomes in elderly care facilities. These could include probiotic cleaning approaches, environmental modification strategies to encourage beneficial microorganisms, and personalized microbial management based on resident health status (94, 95). Additionally, investigating the interactions between the environmental microbiome and the human microbiome in elderly populations would provide valuable insights into microbial transmission dynamics and health implications (92).

In conclusion, our study reveals the complex microbial ecology of premium elderly care facilities, demonstrating how space functionality, environmental parameters, and human factors shape microbial communities across different indoor environments. These findings contribute to our understanding of microbial ecology in built environments and provide a foundation for developing evidence-based strategies to create healthier living environments for elderly populations. By recognizing the ecological complexity of indoor microbiomes and their potential health implications, we can move beyond conventional hygiene approaches toward more nuanced, ecologically informed management strategies that promote both microbial and human health.

### Conclusion

This study provides the first comprehensive analysis of the environmental microbiome across key functional spaces in premium elderly care facilities, revealing critical insights into microbial ecology, environmental drivers, and potential health implications. Our findings demonstrate that microbial community composition and diversity are strongly shaped by space functionality, with dining areas and recreational rooms harboring higher microbial diversity dominated by human-associated and food-related taxa, while medical facilities and bathrooms exhibit lower diversity but higher relative abundance of opportunistic pathogens. Importantly, the effect of functional space (R² = 0.26) exceeded that of geographic location (R² = 0.12) across four cities spanning different climatic zones, indicating that anthropogenic factors associated with space use represent the primary drivers of indoor microbial ecology in elderly care facilities. Environmental parameters, particularly relative humidity and occupancy patterns, emerged as key determinants of microbial community structure, highlighting the importance of indoor environmental management in modulating microbial ecosystems.

The identification of a core microbiome across facilities underscores the existence of stable microbial consortia adapted to elderly care environments, while functional predictions revealed space-specific metabolic potentials, including predicted enrichment of antibiotic resistance genes in medical facilities and carbohydrate metabolism pathways in dining areas. Notably, negative correlations between beneficial bacteria (e.g., *Lactobacillus*) and opportunistic pathogens (e.g., *S. aureus*) observed in relative abundance data are consistent with potential antagonistic interactions, although absolute quantification would be necessary to confirm these ecological relationships and suggest ecological strategies to promote healthier microbial equilibria.

These findings have direct implications for facility management: space-specific hygiene protocols, humidity control, and ventilation strategies may help mitigate pathogen risks while preserving beneficial microbial communities. Furthermore, our results support the potential value of a paradigm shift from pathogen-centric disinfection to ecologically informed microbial stewardship, balancing pathogen reduction with microbiome resilience.

Limitations, including the exclusion of non-bacterial taxa and snapshot sampling, underscore the need for longitudinal, multiomics studies to explore microbial dynamics and host-microbiome interactions. Future research should integrate resident health data to directly link environmental microbiomes to clinical outcomes and test interventions such as probiotic cleaning or bioinformed design. By bridging microbial ecology and geriatric care, this work lays the foundation for creating healthier built environments tailored to the unique needs of aging populations.

## Data Availability

All raw 16S rRNA gene sequencing data have been deposited in the Sequence Read Archive (SRA) of NCBI under the BioProject accession number PRJNA1354094.

## References

[1] United Nations. 2023. Department of economic and social affairs, population division. World population ageing 2023: highlights. UN, New York.

[2] Fang EF, Xie C, Schenkel JA, Wu C, Long Q, Cui H, Aman Y, Frank J, Liao J, Zou H, et al.. 2020. A research agenda for ageing in China in the 21st century (2nd edition): focusing on basic and translational research, long-term care, policy and social networks. Ageing Res Rev 64:101174. doi:10.1016/j.arr.2020.101174 32971255 PMC7505078

[3] National Bureau of Statistics of China. 2024. Statistical communiqué of the People’s Republic of China on the 2023 National Economic and Social Development. Beijing, China

[4] Salazar N, Valdés-Varela L, González S, Gueimonde M, de Los Reyes-Gavilán CG. 2017. Nutrition and the gut microbiome in the elderly. Gut Microbes 8:82–97. doi:10.1080/19490976.2016.1256525 27808595 PMC5390822

[5] Santoro A, Zhao J, Wu L, Carru C, Biagi E, Franceschi C. 2020. Microbiomes other than the gut: inflammaging and age-related diseases. Semin Immunopathol 42:589–605. doi:10.1007/s00281-020-00814-z 32997224 PMC7666274

[6] Gilbert JA, Stephens B. 2018. Microbiology of the built environment. Nat Rev Microbiol 16:661–670. doi:10.1038/s41579-018-0065-5 30127345

[7] Jayaprakash B, Adams RI, Kirjavainen P, Karvonen A, Vepsäläinen A, Valkonen M, Järvi K, Sulyok M, Pekkanen J, Hyvärinen A, Täubel M. 2017. Indoor microbiota in severely moisture damaged homes and the impact of interventions. Microbiome 5:138. doi:10.1186/s40168-017-0356-5 29029638 PMC5640920

[8] Lax S, Sangwan N, Smith D, Larsen P, Handley KM, Richardson M, Guyton K, Krezalek M, Shogan BD, Defazio J, Flemming I, Shakhsheer B, Weber S, Landon E, Garcia-Houchins S, Siegel J, Alverdy J, Knight R, Stephens B, Gilbert JA. 2017. Bacterial colonization and succession in a newly opened hospital. Sci Transl Med 9:eaah6500. doi:10.1126/scitranslmed.aah6500 28539477 PMC5706123

[9] Meadow JF, Altrichter AE, Kembel SW, Kline J, Mhuireach G, Moriyama M, Northcutt D, O’Connor TK, Womack AM, Brown GZ, Green JL, Bohannan BJM. 2014. Indoor airborne bacterial communities are influenced by ventilation, occupancy, and outdoor air source. Indoor Air 24:41–48. doi:10.1111/ina.12047 23621155 PMC4285785

[10] Dannemiller KC, Gent JF, Leaderer BP, Peccia J. 2016. Influence of housing characteristics on bacterial and fungal communities in homes of asthmatic children. Indoor Air 26:179–192. doi:10.1111/ina.12205 25833176 PMC4591094

[11] Rintala H, Pitkäranta M, Täubel M. 2012. Microbial communities associated with house dust. Adv Appl Microbiol 78:75–120. doi:10.1016/B978-0-12-394805-2.00004-X 22305094

[12] Verderber S, Koyabashi U, Dela Cruz C, et al.. 2023. Residential environments for older persons: a comprehensive literature review (2005–2022). Health Environ Res Des J 16:291–337. doi:10.1177/19375867231152611 PMC10328148 37078127

[13] Klepeis NE, Nelson WC, Ott WR, Robinson JP, Tsang AM, Switzer P, Behar JV, Hern SC, Engelmann WH. 2001. The National human activity pattern survey (NHAPS): a resource for assessing exposure to environmental pollutants. J Expo Anal Environ Epidemiol 11:231–252. doi:10.1038/sj.jea.7500165 11477521

[14] Biagi E, Candela M, Fairweather-Tait S, Franceschi C, Brigidi P. 2012. Aging of the human metaorganism: the microbial counterpart. Age (Dordr) 34:247–267. doi:10.1007/s11357-011-9217-5 21347607 PMC3260362

[15] Adams RI, Bateman AC, Bik HM, Meadow JF. 2015. Microbiota of the indoor environment: a meta-analysis. Microbiome 3:49. doi:10.1186/s40168-015-0108-3 26459172 PMC4604073

[16] Fahimipour AK, Ben Mamaar S, McFarland AG, Blaustein RA, Chen J, Glawe AJ, Kline J, Green JL, Halden RU, Van Den Wymelenberg K, Huttenhower C, Hartmann EM. 2018. Antimicrobial chemicals associate with microbial function and antibiotic resistance indoors. mSystems 3:e00200-18. doi:10.1128/mSystems.00200-18 30574558 PMC6290264

[17] Stefanini I, Cavalieri D. 2018. Metagenomic approaches to investigate the contribution of the vineyard environment to the quality of wine fermentation: potentials and difficulties. Front Microbiol 9:991. doi:10.3389/fmicb.2018.00991 29867889 PMC5964215

[18] Hoisington AJ, Brenner LA, Kinney KA, Postolache TT, Lowry CA. 2015. The microbiome of the built environment and mental health. Microbiome 3:60. doi:10.1186/s40168-015-0127-0 26674771 PMC4682225

[19] Montoya A, Mody L. 2011. Common infections in nursing homes: a review of current issues and challenges. Aging health 7:889–899. doi:10.2217/AHE.11.80 23264804 PMC3526889

[20] Roghmann MC, Johnson JK, Sorkin JD, et al.. 2015. Transmission of MRSA to healthcare personnel gowns and gloves during care of nursing home residents. Infect Control Hosp Epidemiol 36:1050–1057. doi:10.1017/ice.2015.119 26008727 PMC4900177

[21] Montoya A, Cassone M, Mody L. 2016. Infections in nursing homes: epidemiology and prevention programs. Clin Geriatr Med 32:585–607. doi:10.1016/j.cger.2016.02.004 27394025

[22] Lax S, Gilbert JA. 2015. Hospital-associated microbiota and implications for nosocomial infections. Trends Mol Med 21:427–432. doi:10.1016/j.molmed.2015.03.005 25907678

[23] Cristina ML, Spagnolo AM, Giribone L, et al.. 2021. Epidemiology and prevention of healthcare-associated infections in geriatric patients: a narrative review. Int J Environ Res Public Health 18:5333. doi:10.3390/ijerph18105333 34067797 PMC8156303

[24] National Health Commission of China. 2020. National standard for environmental hygiene in elderly care facilities (GB38600-2019). Beijing, China

[25] Ramos T, Stephens B. 2014. Tools to improve built environment data collection for indoor microbial ecology investigations. Build Environ 81:243–257. doi:10.1016/j.buildenv.2014.07.004

[26] Jo J, Oh J, Park C. 2020. Microbial community analysis using high-throughput sequencing technology: a beginner’s guide for microbiologists. J Microbiol 58:176–192. doi:10.1007/s12275-020-9525-5 32108314

[27] Adams RI, Bhangar S, Pasut W, Arens EA, Taylor JW, Lindow SE, Nazaroff WW, Bruns TD. 2015. Chamber bioaerosol study: outdoor air and human occupants as sources of indoor airborne microbes. PLoS One 10:e0128022. doi:10.1371/journal.pone.0128022 26024222 PMC4449033

[28] Dannemiller KC, Weschler CJ, Peccia J. 2017. Fungal and bacterial growth in floor dust at elevated relative humidity levels. Indoor Air 27:354–363. doi:10.1111/ina.12313 27272645

[29] Leung MHY, Lee PKH. 2016. The roles of the outdoors and occupants in contributing to a potential pan-microbiome of the built environment: a review. Microbiome 4:21. doi:10.1186/s40168-016-0165-2 27216717 PMC4877933

[30] Klindworth A, Pruesse E, Schweer T, Peplies J, Quast C, Horn M, Glöckner FO. 2013. Evaluation of general 16S ribosomal RNA gene PCR primers for classical and next-generation sequencing-based diversity studies. Nucleic Acids Res 41:e1. doi:10.1093/nar/gks808 22933715 PMC3592464

[31] Meisel JS, Hannigan GD, Tyldsley AS, SanMiguel AJ, Hodkinson BP, Zheng Q, Grice EA. 2016. Skin microbiome surveys are strongly influenced by experimental design. J Invest Dermatol 136:947–956. doi:10.1016/j.jid.2016.01.016 26829039 PMC4842136

[32] Caporaso JG, Lauber CL, Walters WA, Berg-Lyons D, Huntley J, Fierer N, Owens SM, Betley J, Fraser L, Bauer M, Gormley N, Gilbert JA, Smith G, Knight R. 2012. Ultra-high-throughput microbial community analysis on the Illumina HiSeq and MiSeq platforms. ISME J 6:1621–1624. doi:10.1038/ismej.2012.8 22402401 PMC3400413

[33] Bolyen E, Rideout JR, Dillon MR, Bokulich NA, Abnet CC, Al-Ghalith GA, Alexander H, Alm EJ, Arumugam M, Asnicar F, et al.. 2019. Reproducible, interactive, scalable and extensible microbiome data science using QIIME 2. Nat Biotechnol 37:852–857. doi:10.1038/s41587-019-0209-9 31341288 PMC7015180

[34] Callahan BJ, McMurdie PJ, Rosen MJ, Han AW, Johnson AJA, Holmes SP. 2016. DADA2: high-resolution sample inference from Illumina amplicon data. Nat Methods 13:581–583. doi:10.1038/nmeth.3869 27214047 PMC4927377

[35] Quast C, Pruesse E, Yilmaz P, Gerken J, Schweer T, Yarza P, Peplies J, Glöckner FO. 2013. The SILVA ribosomal RNA gene database project: improved data processing and web-based tools. Nucleic Acids Res 41:D590–6. doi:10.1093/nar/gks1219 23193283 PMC3531112

[36] Lozupone C, Knight R. 2005. UniFrac: a new phylogenetic method for comparing microbial communities. Appl Environ Microbiol 71:8228–8235. doi:10.1128/AEM.71.12.8228-8235.2005 16332807 PMC1317376

[37] Mandal S, Van Treuren W, White RA, Eggesbø M, Knight R, Peddada SD. 2015. Analysis of composition of microbiomes: a novel method for studying microbial composition. Microb Ecol Health Dis 26:27663. doi:10.3402/mehd.v26.27663 26028277 PMC4450248

[38] Douglas GM, Maffei VJ, Zaneveld JR, Yurgel SN, Brown JR, Taylor CM, Huttenhower C, Langille MGI. 2020. PICRUSt2 for prediction of metagenome functions. Nat Biotechnol 38:685–688. doi:10.1038/s41587-020-0548-6 32483366 PMC7365738

[39] Liu B, Zheng D, Zhou S, Chen L, Yang J. 2022. VFDB 2022: a general classification scheme for bacterial virulence factors. Nucleic Acids Res 50:D912–D917. doi:10.1093/nar/gkab1107 34850947 PMC8728188

[40] Team RC. 2021. R: a language and environment for statistical computing. R Foundation for Statistical Computing, Vienna, Austria.

[41] McMurdie PJ, Holmes S. 2013. Phyloseq: an R package for reproducible interactive analysis and graphics of microbiome census data. PLoS One 8:e61217. doi:10.1371/journal.pone.0061217 23630581 PMC3632530

[42] Oksanen J, Blanchet FG, Friendly M, et al.. 2022. Vegan: community ecology package. R package version 2.6-2. Available from: http://cran.r-project.org

[43] Love MI, Huber W, Anders S. 2014. Moderated estimation of fold change and dispersion for RNA-seq data with DESeq2. Genome Biol 15. doi:10.1186/s13059-014-0550-8 PMC4302049 25516281

[44] Anderson MJ. 2001. A new method for non-parametric multivariate analysis of variance. Austral Ecol 26:32–46. doi:10.1046/j.1442-9993.2001.01070.x

[45] Legendre P, Anderson MJ. 1999. Distance-based redundancy analysis: testing multispecies responses in multifactorial ecological experiments. Ecol Monogr 69:1–24. doi:10.1890/0012-9615(1999)069[0001:DBRATM]2.0.CO;2

[46] Kurtz ZD, Müller CL, Miraldi ER, Littman DR, Blaser MJ, Bonneau RA. 2015. Sparse and compositionally robust inference of microbial ecological networks. PLoS Comput Biol 11:e1004226. doi:10.1371/journal.pcbi.1004226 25950956 PMC4423992

[47] Csardi G, Nepusz T. 2006. The igraph software package for complex network research. InterJ Complex Syst 1695:1–9.

[48] Chase J, Fouquier J, Zare M, Sonderegger DL, Knight R, Kelley ST, Siegel J, Caporaso JG. 2016. Geography and location are the primary drivers of office microbiome composition. mSystems 1:e00022-16. doi:10.1128/mSystems.00022-16 27822521 PMC5069741

[49] Kembel SW, Jones E, Kline J, Northcutt D, Stenson J, Womack AM, Bohannan BJM, Brown GZ, Green JL. 2012. Architectural design influences the diversity and structure of the built environment microbiome. ISME J 6:1469–1479. doi:10.1038/ismej.2011.211 22278670 PMC3400407

[50] Stephens B, Azimi P, Thoemmes MS, Heidarinejad M, Allen JG, Gilbert JA. 2019. Microbial exchange via fomites and implications for human health. Curr Pollution Rep 5:198–213. doi:10.1007/s40726-019-00123-6 PMC7149182 34171005

[51] National Academies of Sciences, Engineering, and Medicine. 2017. Microbiomes of the built environment: a research agenda for indoor microbiology, human health, and buildings. The National Academies Press, Washington, DC. 29035489

[52] Peccia J, Kwan SE. 2016. Buildings, beneficial microbes, and health. Trends Microbiol 24:595–597. doi:10.1016/j.tim.2016.04.007 27397930

[53] Lax S, Smith DP, Hampton-Marcell J, Owens SM, Handley KM, Scott NM, Gibbons SM, Larsen P, Shogan BD, Weiss S, Metcalf JL, Ursell LK, Vázquez-Baeza Y, Van Treuren W, Hasan NA, Gibson MK, Colwell R, Dantas G, Knight R, Gilbert JA. 2014. Longitudinal analysis of microbial interaction between humans and the indoor environment. Science 345:1048–1052. doi:10.1126/science.1254529 25170151 PMC4337996

[54] Fahimipour AK, Hartmann EM, Siemens A, Kline J, Levin DA, Wilson H, Betancourt-Román CM, Brown GZ, Fretz M, Northcutt D, Siemens KN, Huttenhower C, Green JL, Van Den Wymelenberg K. 2018. Daylight exposure modulates bacterial communities associated with household dust. Microbiome 6:175. doi:10.1186/s40168-018-0559-4 30333051 PMC6193304

[55] Gibbons SM. 2016. The built environment is a microbial wasteland. mSystems 1. doi:10.1128/mSystems.00033-16 PMC5069742 27832216

[56] Oberauner L, Zachow C, Lackner S, Högenauer C, Smolle K-H, Berg G. 2013. The ignored diversity: complex bacterial communities in intensive care units revealed by 16S pyrosequencing. Sci Rep 3:1413. doi:10.1038/srep01413 23475210 PMC3593336

[57] Lax S, Cardona C, Zhao D, Winton VJ, Goodney G, Gao P, Gottel N, Hartmann EM, Henry C, Thomas PM, Kelley ST, Stephens B, Gilbert JA. 2019. Microbial and metabolic succession on common building materials under high humidity conditions. Nat Commun 10:1767. doi:10.1038/s41467-019-09764-z 30992445 PMC6467912

[58] Hartmann EM, Hickey R, Hsu T, Betancourt Román CM, Chen J, Schwager R, Kline J, Brown GZ, Halden RU, Huttenhower C, Green JL. 2016. Antimicrobial chemicals are associated with elevated antibiotic resistance genes in the indoor dust microbiome. Environ Sci Technol 50:9807–9815. doi:10.1021/acs.est.6b00262 27599587 PMC5032049

[59] Prussin AJ II, Marr LC. 2015. Sources of airborne microorganisms in the built environment. Microbiome 3:78. doi:10.1186/s40168-015-0144-z 26694197 PMC4688924

[60] Hu J, Ben Maamar S, Glawe AJ, Gottel N, Gilbert JA, Hartmann EM. 2019. Impacts of indoor surface finishes on bacterial viability. Indoor Air 29:551–562. doi:10.1111/ina.12558 30980566 PMC6851865

[61] Fu X, Li Y, Yuan Q, Cai G, Deng Y, Zhang X, Norbäck D, Sun Y. 2020. Continental-scale microbiome study reveals different environmental characteristics determining microbial richness, composition, and quantity in hotel rooms. mSystems 5. doi:10.1128/msystems.00119-20 PMC7253364 32430405

[62] Adams RI, Lymperopoulou DS, Misztal PK, De Cassia Pessotti R, Behie SW, Tian Y, Goldstein AH, Lindow SE, Nazaroff WW, Taylor JW, Traxler MF, Bruns TD. 2017. Microbes and associated soluble and volatile chemicals on periodically wet household surfaces. Microbiome 5:128. doi:10.1186/s40168-017-0347-6 28950891 PMC5615633

[63] Liu Y, Misztal PK, Xiong J, Tian Y, Arata C, Weber RJ, Nazaroff WW, Goldstein AH. 2019. Characterizing sources and emissions of volatile organic compounds in a northern California residence using space‐ and time‐resolved measurements. Indoor Air 29:630–644. doi:10.1111/ina.12562 31004537

[64] Hospodsky D, Qian J, Nazaroff WW, Yamamoto N, Bibby K, Rismani-Yazdi H, Peccia J. 2012. Human occupancy as a source of indoor airborne bacteria. PLoS One 7:e34867. doi:10.1371/journal.pone.0034867 22529946 PMC3329548

[65] Leung MHY, Wilkins D, Li EKT, Kong FKF, Lee PKH. 2014. Indoor-air microbiome in an urban subway network: diversity and dynamics. Appl Environ Microbiol 80:6760–6770. doi:10.1128/AEM.02244-14 25172855 PMC4249038

[66] Flores GE, Bates ST, Knights D, Lauber CL, Stombaugh J, Knight R, Fierer N. 2011. Microbial biogeography of public restroom surfaces. PLoS One 6:e28132. doi:10.1371/journal.pone.0028132 22132229 PMC3223236

[67] Faust K, Raes J. 2012. Microbial interactions: from networks to models. Nat Rev Microbiol 10:538–550. doi:10.1038/nrmicro2832 22796884

[68] Bowers RM, McLetchie S, Knight R, Fierer N. 2011. Spatial variability in airborne bacterial communities across land-use types and their relationship to the bacterial communities of potential source environments. ISME J 5:601–612. doi:10.1038/ismej.2010.167 21048802 PMC3105744

[69] Chen Y, Fu X, Ou Z, Li J, Lin S, Wu Y, Wang X, Deng Y, Sun Y. 2023. Environmental determinants and demographic influences on global urban microbiomes, antimicrobial resistance and pathogenicity. NPJ Biofilms Microbiomes 9:94. doi:10.1038/s41522-023-00459-4 38062054 PMC10703778

[70] Richardson M, Gottel N, Gilbert JA, Gordon J, Gandhi P, Reboulet R, Hampton-Marcell JT. 2019. Concurrent measurement of microbiome and allergens in the air of bedrooms of allergy disease patients in the Chicago area. Microbiome 7:82. doi:10.1186/s40168-019-0695-5 31159879 PMC6547563

[71] Ramos T, Dedesko S, Siegel JA, Gilbert JA, Stephens B. 2015. Spatial and temporal variations in indoor environmental conditions, human occupancy, and operational characteristics in a new hospital building. PLoS One 10:e0118207. doi:10.1371/journal.pone.0118207 25729898 PMC4346405

[72] Aguiar-Pulido V, Huang W, Suarez-Ulloa V, Cickovski T, Mathee K, Narasimhan G. 2016. Metagenomics, metatranscriptomics, and metabolomics approaches for microbiome analysis. Evol Bioinform Online 12:5–16. doi:10.4137/EBO.S36436 27199545 PMC4869604

[73] Fujimura KE, Demoor T, Rauch M, Faruqi AA, Jang S, Johnson CC, Boushey HA, Zoratti E, Ownby D, Lukacs NW, Lynch SV. 2014. House dust exposure mediates gut microbiome Lactobacillus enrichment and airway immune defense against allergens and virus infection. Proc Natl Acad Sci USA 111:805–810. doi:10.1073/pnas.1310750111 24344318 PMC3896155

[74] Tun MH, Tun HM, Mahoney JJ, Konya TB, Guttman DS, Becker AB, Mandhane PJ, Turvey SE, Subbarao P, Sears MR, Brook JR, Lou W, Takaro TK, Scott JA, Kozyrskyj AL. 2018. Postnatal exposure to household disinfectants, infant gut microbiota and subsequent risk of overweight in children. CMAJ 190:E1097–E1107. doi:10.1503/cmaj.170809 30224442 PMC6141245

[75] Fu X, Ou Z, Sun Y. 2022. Indoor microbiome and allergic diseases: from theoretical advances to prevention strategies. Eco Environ Health 1:133–146. doi:10.1016/j.eehl.2022.09.002 38075599 PMC10702906

[76] O’Toole PW, Jeffery IB. 2015. Gut microbiota and aging. Science 350:1214–1215. doi:10.1126/science.aac8469 26785481

[77] Vandegrift R, Bateman AC, Siemens KN, Nguyen M, Wilson HE, Green JL, Van Den Wymelenberg KG, Hickey RJ. 2017. Cleanliness in context: reconciling hygiene with a modern microbial perspective. Microbiome 5:76. doi:10.1186/s40168-017-0294-2 28705228 PMC5513348

[78] Dannemiller KC. 2019. Moving towards a robust definition for a “healthy” indoor microbiome. mSystems 4:e00074-19. doi:10.1128/mSystems.00074-19 31120023 PMC6529541

[79] Zhu B, Wang X, Li L. 2010. Human gut microbiome: the second genome of human body. Protein Cell 1:718–725. doi:10.1007/s13238-010-0093-z 21203913 PMC4875195

[80] Brown GZ, Kline J, Mhuireach G, Northcutt D, Stenson J. 2016. Making microbiology of the built environment relevant to design. Microbiome 4:6. doi:10.1186/s40168-016-0152-7 26880354 PMC4754988

[81] Green JL. 2014. Can bioinformed design promote healthy indoor ecosystems? Indoor Air 24:113–115. doi:10.1111/ina.12090 24628783

[82] Xie J, Jin L, Luo X, Zhao Z, Li X. 2018. Seasonal disparities in airborne bacteria and associated antibiotic resistance genes in PM _2.5_ between urban and rural sites. Environ Sci Technol Lett 5:74–79. doi:10.1021/acs.estlett.7b00561

[83] Li H, Zhou X-Y, Yang X-R, Zhu Y-G, Hong Y-W, Su J-Q. 2019. Spatial and seasonal variation of the airborne microbiome in a rapidly developing city of China. Science of The Total Environment 665:61–68. doi:10.1016/j.scitotenv.2019.01.367 30772579

[84] Ying S, Zeng D-N, Chi L, Tan Y, Galzote C, Cardona C, Lax S, Gilbert J, Quan Z-X. 2015. The influence of age and gender on skin-associated microbial communities in urban and rural human populations. PLoS One 10:e0141842. doi:10.1371/journal.pone.0141842 26510185 PMC4624872

[85] Biagi E, Nylund L, Candela M, Ostan R, Bucci L, Pini E, Nikkïla J, Monti D, Satokari R, Franceschi C, Brigidi P, De Vos W. 2010. Through ageing, and beyond: gut microbiota and inflammatory status in seniors and centenarians. PLoS One 5:e10667. doi:10.1371/journal.pone.0010667 20498852 PMC2871786

[86] Gilbert JA, Blaser MJ, Caporaso JG, Jansson JK, Lynch SV, Knight R. 2018. Current understanding of the human microbiome. Nat Med 24:392–400. doi:10.1038/nm.4517 29634682 PMC7043356

[87] Amend AS, Seifert KA, Samson R, Bruns TD. 2010. Indoor fungal composition is geographically patterned and more diverse in temperate zones than in the tropics. Proc Natl Acad Sci USA 107:13748–13753. doi:10.1073/pnas.1000454107 20616017 PMC2922287

[88] Thompson LR, Sanders JG, McDonald D, Amir A, Ladau J, Locey KJ, Prill RJ, Tripathi A, Gibbons SM, Ackermann G, et al.. 2017. A communal catalogue reveals Earth’s multiscale microbial diversity. Nature 551:457–463. doi:10.1038/nature24621 29088705 PMC6192678

[89] Gall-David S, Boudry G, Buffet-Bataillon S, et al.. 2023. Comparison of Four DNA Extraction Kits Efficiency for 16SrDNA Microbiota Profiling of Diverse Human Samples. Future Sci. OA 9:FOS837. doi:10.2144/fsoa-2022-0072 PMC10051199 37006230

[90] Claesson MJ, Jeffery IB, Conde S, Power SE, O’Connor EM, Cusack S, Harris HMB, Coakley M, Lakshminarayanan B, O’Sullivan O, et al.. 2012. Gut microbiota composition correlates with diet and health in the elderly. Nature 488:178–184. doi:10.1038/nature11319 22797518

[91] Jiao P, Jiang Y, Jiao J, et al.. 2021. The pathogenic characteristics and influencing factors of health care-associated infection in elderly care center under the mode of integration of medical care and elderly care service: a cross-sectional study. Medicine (Abingdon) 100:e26158. doi:10.1097/MD.0000000000026158 PMC8154447 34032774

[92] Gweon HS, Shaw LP, Swann J, De Maio N, AbuOun M, Niehus R, Hubbard ATM, Bowes MJ, Bailey MJ, Peto TEA, Hoosdally SJ, Walker AS, Sebra RP, Crook DW, Anjum MF, Read DS, Stoesser N. 2019. The impact of sequencing depth on the inferred taxonomic composition and AMR gene content of metagenomic samples. Environ Microbiome 14:7. doi:10.1186/s40793-019-0347-1 33902704 PMC8204541

[93] Dunn RR, Fierer N, Henley JB, Leff JW, Menninger HL. 2013. Home life: factors structuring the bacterial diversity found within and between homes. PLoS One 8:e64133. doi:10.1371/journal.pone.0064133 23717552 PMC3661444

[94] Sharma A, Gilbert JA. 2018. Microbial exposure and human health. Curr Opin Microbiol 44:79–87. doi:10.1016/j.mib.2018.08.003 30195150

[95] Caselli E, Brusaferro S, Coccagna M, Arnoldo L, Berloco F, Antonioli P, Tarricone R, Pelissero G, Nola S, La Fauci V, Conte A, Tognon L, Villone G, Trua N, Mazzacane S. 2018. Reducing healthcare-associated infections incidence by a probiotic-based sanitation system: a multicentre, prospective, intervention study. PLoS One 13:e0199616. doi:10.1371/journal.pone.0199616 30001345 PMC6042698

